# Comprehensive Evaluation of Metal Pollution in Urban Soils of a Post-Industrial City—A Case of Łódź, Poland

**DOI:** 10.3390/molecules25184350

**Published:** 2020-09-22

**Authors:** Kinga Wieczorek, Anna Turek, Małgorzata Szczesio, Wojciech M. Wolf

**Affiliations:** Institute of General and Ecological Chemistry, Lodz University of Technology, 116 Żeromskiego Str., 90–924 Lodz, Poland; malgorzata.szczesio@p.lodz.pl (M.S.); wojciech.wolf@p.lodz.pl (W.M.W.)

**Keywords:** urban soil, metal pollution, hot-spots, GIS, multivariate analysis

## Abstract

The pollution of urban soils by metals is a global problem. Prolonged exposure of habitants who are in contact with metals retained in soil poses a health risk. This particularly applies to industrialized cities with developed transport networks. The aim of the study was to determine the content and spatial distribution of mobile metal fractions in soils of the city of Łódź and to identify their load and sources. Multivariate statistical analysis (principal component analysis (PCA), cluster analysis (CA)), combined with GIS, were used to make a comprehensive evaluation of the soil contamination. Hot-spots and differences between urban and suburban areas were also investigated. Metals were determined by atomic absorption spectrometry (AAS) after soil extraction with 1 mol L^−1^ HCl. In most sites, the metal content changes in the following order: Zn > Pb > Cu > Ni > Cd. About one-third of the samples are considerably (or very highly) contaminated, (contamination factor, CF > 3) with Cu, Pb, or Zn. In almost 40% of the samples, contaminated soils were found (pollution load index, PLI > 1). All metals have a strong influence on the first principal component (PC1), whereas second principal component (PC2) is related to pH. Polluted soils are located in the downtown, in the south and east part of the city. The distribution of contamination coincides with the urban layout, low emission sources and former industrial areas of Łódź.

## 1. Introduction

Nowadays, soil contamination attributed mainly to intense industrialization, urbanization and transport is becoming a global issue [1,2,3,4]. Soil pollution by potentially toxic elements represents one of the problems for the sustainable development of urban areas. The Soil Genesis, Classification and Cartography Commission of the Soil Science Society of Poland noticed this problem and, therefore, announced Technosol as Soil of the Year 2020 [5]. Technosols are defined as soils strongly influenced by human activity and modified by technical processes (often referred to as urban and industrial areas). They are characterized by high contents of artefacts in their profile. Urban soil is the sink of hazardous substances, e.g., metals. Since metals are omnipresent, their adverse effects on human health are very likely. Metals, due to their long biological half-lives and high toxicity, tend to accumulate in the environment. The persistence of potentially toxic elements in soil is much longer than in other compartments of the environment [6,7]. It is estimated that the metal load of the anthropogenic origin often outweighs the load from geogenic and pedogenic sources. In addition, metals from anthropogenic processes are usually more mobile and bioavailable [7]. Urban soils are largely artificially developed or contain admixtures of contaminated materials. They are developed as a result of strong human influences and are characterized by diverse properties, depending on the origin and composition of the parent and anthropogenic materials. Soils differ in their abundance and degree of pollution. Urban soil could be frequently disturbed and resuspended due to dense population and heavy traffic, so the people in urban regions could easily be exposed to metals in the soil via three routes: direct dermal contact or dust inhalation and indirect input as a result of ingestion (in the form of contaminated food products). Roadside deposited dust from traffic emissions (as well as from the combustion of solid fuels) is of particular concern as being a carrier of potentially toxic elements, as it may pose a health risk [7,8,9,10]. Metals in urban soils are considered as very useful indicators of environmental pollution [10,11].

Metal concentrations in urban topsoil are strongly influenced by land use, e.g., residential, commercial, industrial, recreational, and even agricultural functions [12]. Therefore, intervention criteria to assess the pollution of the soil surface are defined in relation to the type of land use. Anthropogenic activities cause disturbance and heterogeneity of soil. As a result, young soils with unpredictable layering and limited organic matter content are formed. At the initial stage of soil development, metals are not tightly bound to the matrix. Therefore, they are more mobile and available to plants. 

The behavior of potentially toxic elements in the environment, their bioavailability, and toxicity are more related to the forms in which they occur in the soil than to their total content. However, the total metal content is usually taken as the criterion for soil contamination assessment, regardless of their actual availability [7,13,14]. In making decisions aimed at reducing soil pollution and the improvement of soil functions, it is critical to analyze the content of mobile metal fractions in soil [15]. Therefore, the development of a simple and quick method for assessing the quality of urban soils is a key issue.

The analysis of metal content in urban soils is the subject of numerous publications [7,8,9,10,11,12,13,14,15,16,17,18,19,20]. Particular attention is paid to green areas due to their use for recreation and the possibility of people’s exposure to contact with contaminated soil [2,9,21,22,23]. The abundance of several urban soils meets the requirements for arable soils, and they can be used for cultivation. Allotment gardens situated in the city are a special type of cultivated area and are often created on brownfields or in the vicinity of roads. Besides industry, traffic, and urban waste, unreasonable and (usually) excessive use of fertilizers and pesticides frequently causes additional pollution of soil—much higher than that of typically cultivated areas. Growing plants on such soil is another threat to human health. For this reason, regular monitoring of both food and soil is necessary to reduce the exposure of consumers to ingestion of metals [7,24,25,26,27].

Reports from European countries indicate that metals are one of the most frequent (37.3%) soil contaminants. The polluting activities vary considerably across Europe, but the most serious sources are industry and waste disposal. Remediation of polluted sites is complex and expensive in particular when old historical contamination is concerned. Although most European countries apply legislation “polluter-pays”, many of the responsible polluters no longer exist or are insolvent [28]. According to the Soil Science Society of Poland, about 60% of Polish citizens live in areas dominated by technogenic soils (i.e., Technosols—World Reference Base for Soil Resources (WRB) classification system) [5,29]. Particularly, for this reason, it is necessary to monitor them regularly. Unfortunately, in Poland there are no provisions regarding the permissible content of mobile fractions of metals in soils to date. Polish law [30] defines certain thresholds of the total metal content in soils for various types of use. 

This paper presents investigations on the content of mobile fractions of cadmium, copper, lead, nickel, and zinc in urban soils. Łódź was chosen as an important Polish agglomeration with a specific history of the city evolution. Łódź is a post-industrial city, which emerged as a result of the rapid industry development (especially textile manufacturing), which took place at the beginning of the 19th century. The dynamic industrialization process and substantial growth of population made Łódź a world-scale phenomenon. However, it gave rise to numerous negative consequences, which, until today, affect the natural environment and spatial structure of the city. Large textile and clothing factories collapsed in the early 1990s. The majority of the production was transferred to various smaller enterprises, which rose up either from the old mills or new, green sites. Nevertheless, substantial metal pollution generated during the industrial period remained in the central and southern part of the city. After 1945, many villages located in the vicinity of Łódź were incorporated within the city boundaries. This change was reflected in the spatial management of Łódź, because the urban area spread outside the ring of the circular railway. The large new residential areas isolated from one another, spatially, were mainly raised in the eastern and western part of the city, and created the outskirts [31,32,33,34].

From 1988 to 2010, metal contamination of soils at selected sites in Łódź was the subject of several works [35,36,37,38,39,40,41,42]. Allotment garden areas and the impact of three power plants operating in the city were examined. It was found that the limits of metal content in soils set for industrial areas and gardens were not exceeded, but in both cases, the dominant share of metals from road transport was confirmed. 

The aims of this study comprise: (1) determination and spatial distribution of mobile fraction of Zn, Cu, Pb, Ni, Cd contents, and pH in the surface soil of post-industrial city; (2) evaluation of the pollution levels for individual variable by contamination factor (CF) and individual sampling points by pollution load index (PLI); (3) application of a comprehensive visualization method based on principal component analysis (PCA), cluster analysis (CA), and geographic information system (GIS) for interpretation of geochemistry data in the city characterized by the multi-anthropogenic pollution sources; (4) identification of hot-spots with outlying metals concentration, pH values, and determination of possible origins of pollutants; (5) investigation of the differences between the impact of anthropopressure on urban and suburban areas.

## 2. Results and Discussion

### 2.1. Descriptive Statistic, Pollution Indices, Single-Metal Distribution Maps

**Figure 1:**
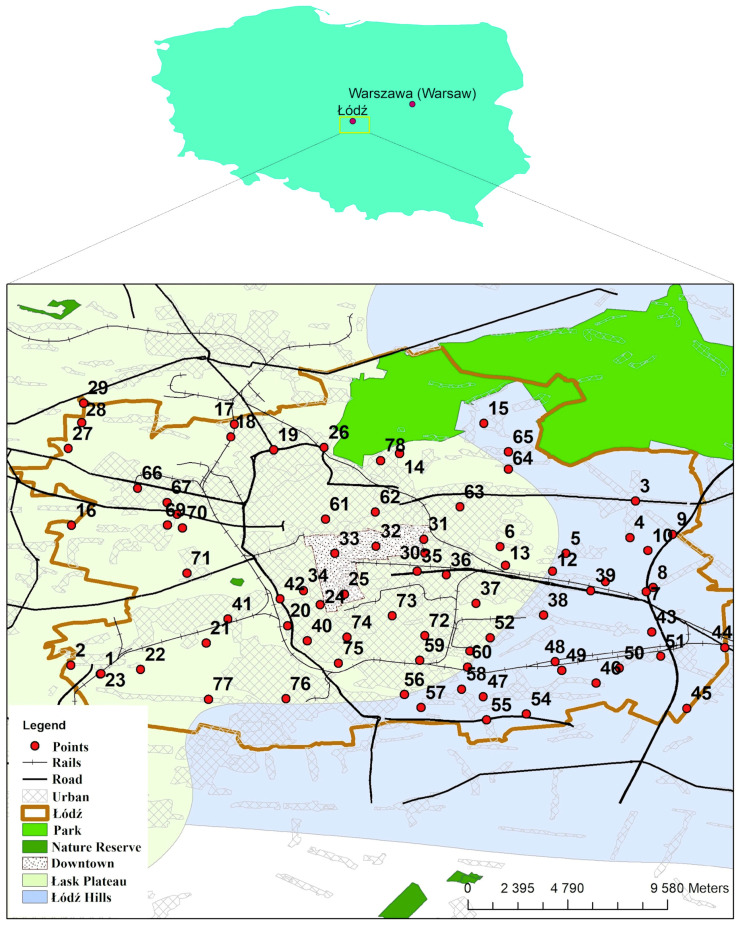
Location of the sampling sites in Łódź (Poland).

**Figure 2:**
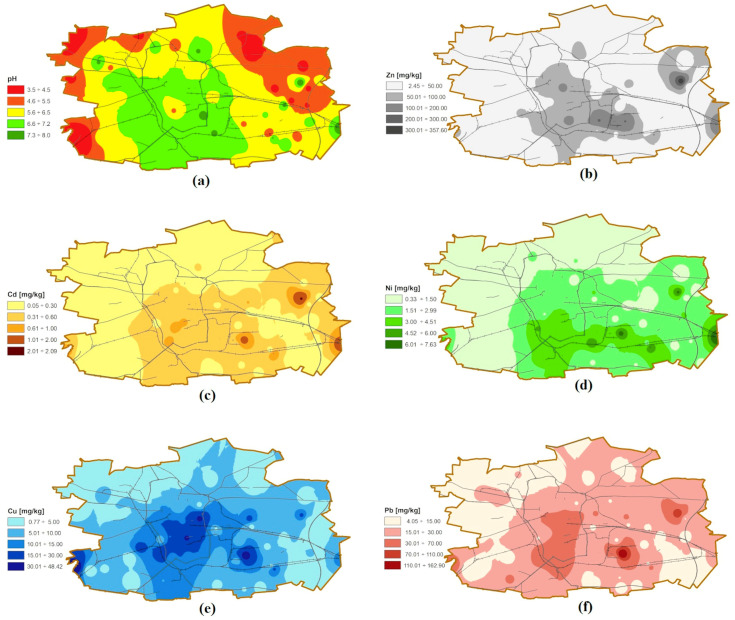
Spatial distribution of (**a**) pH and mobile fractions of: (**b**) Zn; (**c**) Cd; (**d**) Ni; (**e**) Cu; (**f**) Pb in soil.

The studied samples (Figure 1) belong to mineral soils with organic matter content below 10% (Table S1, Supplementary Material). Sand was a dominant textural class (54 samples), soils in the west and northeast parts of the city were classified as loamy sand (13 samples) and sandy loam (11 samples) [43]—Table S1. Among 78 sampling sites, more than 53% were very acidic (pH < 4.5), acidic (4.6–5.5), or slightly acidic (5.6–6.5). Over 85% samples collected from agricultural areas represent pH < 6.6. Neutral (pH 6.6–7.2) and alkaline soils occur in the city center and in the southern part of Łódź, primarily in urbanized areas (old town tenement houses, large housing estates)—Figure 2a, Table S1. Metal contents are in Table S2. The percentage share of metal mobile fractions as extracted by 1 mol L^−1^ HCl within the total metal contents varies for particular metal and soil samples: 2.17–29.8% for Ni, 5.19–85.2% for Cd, 3.08–90.9% for Zn, 6.30–96.7% for Cu, and 8.21–88.7% for Pb. The average values are 12.6%, 32.8%, 36.4%, 52.5%, and 57.8%, respectively. High Spearman correlations (*p* < 0.01) between mobile and total metal contents were observed for Zn (0.86), for Cu (0.85), and for Pb (0.84), and moderate for Ni (0.68). Weak relationship was detected for Cd (0.31). As pointed out by [7,13,14,15], total metal contents are not sufficient to assess their potential toxicity. Therefore, further considerations are based on mobile fractions only.

To assess the soil pollution, the extraction of metal mobile fractions was performed in 1 mol L^−1^ HCl solution [15,44,45,46,47], since following the comprehensive studies [7,15,44,47], HCl does not destroy silicates matrix. Therefore, extraction with diluted HCl is a more reliable method for the determination of bioavailable fractions than an estimation based on the total metals content. Moreover, a strong correlation between metal contents accumulated in plants and extracted from the soil was recognized. HCl extracts the mobile phases of metals (water soluble, exchangeable) but also binds with carbonates. The results are summarized in Table 1, and indicate that metal contents are not normally distributed. This observation is supported by the Kolmogorov–Smirnov (K–S) test (*p* value < 0.05), high values of skewness, and kurtosis coefficients. Skewness values are larger than 1, which show that distribution asymmetry is right-skewed for all metals [48]. It is consistent with mean, median, and upper quartile. Most of the results are lower than their means, which, in turn, are higher than median values [49]. The most and least symmetrical distributions are observed for Ni and Pb, respectively. Asymmetrical distribution may indicate the anthropogenic origin of metals [50]. Kurtosis values are higher than 0 for all metals. It indicates leptokurtic distribution, which is characterized by being more peaked shape than normal [17,48,51]. The best fitting for all metals is presented by lognormal distribution (K–S *p* value > 0.20) [8]. Coefficient of variations (CV) also confirmed unsymmetrical distribution of variables. CV values exceed 100% for Zn, Cu, and Pb, which show exceptionally high variability. Cd and Ni are characterized by high variability (ranged from 51% to 100%) [52]. High CV values indicate the influence of numerous point sources of pollutants. Spatial metal distributions are shown in Figure 2b–f.

According to the mean and median, the metal contents follow a decreasing order: Zn > Pb > Cu > Ni > Cd. Similar relationships were obtained in Vancouver soils [7] and in Hungarian cities [14]. However, some exceptions are observed for individual points. Pb contents are higher than Zn ones for 24 points (Figure S1) that are located beyond the city center, mainly in its northern part. It is presumably due to the various origins or sorption affinity of Pb and Zn onto the soil components [53]. Most of these samples belong to agricultural soils and wastelands with a predominance of a very acidic and acidic reaction. Arable soils, grasslands, and wastelands usually contain more humic substances compared to Technosols. Pb has an affinity for organic compounds, forming less mobile high-molecular-weight organo-complexes [27]. Furthermore, although pH is a critical parameter for metal mobility [7,27,54], long-term retention of metals in soil causes their binding in less soluble forms. Generally, metal mobility increases with soil acidity. However, according to Hou et al. [55], the Pb content in soils is sometimes negatively correlated with pH.

Skewness coefficient of pH suggests normal distribution [56], but kurtosis values are different from zero. Following the results of the K–S test, pH values are not normally distributed. However, the shape of this distribution is different from that of the metals. Negative skewness indicates slightly left-skewed asymmetry, which is confirmed by mean lower than median. Kurtosis values are also negative, hence the distribution is platykurtic. According to shape parameters, pH distribution is more flattened than normal, and a minor part of the results is higher than mean. Soil pH did not pass the K–S test for either of the feasible distributions. Two peaks in the histogram are observed, which suggest bimodal distribution. Unlike metals, soil reaction is associated with non-point sources (low variability is confirmed by CV value [52]).

According to Polish Standards [45,46], the agricultural soil classification includes three classes of abundance (soil abundance is defined as the content of micro- and macroelements available to plants). Soil from class I is characterized by high abundance, which means that it does not require fertilization. Soil that belongs to class II (moderate abundance) requires fertilization only for plants sensitive to nutrient deficiency. Soil of class III should be intensively amended with nutrients. In each class, the content of nutrient depends on the agronomic category. Agronomic categories of soils are determined based on the texture classification. For soil containing up to 10% of the fraction <0.02 mm (agronomic category—very light soil [57]), the limit values of the elements in soil are: for class I > 3.3 mg kg^−1^ (Zn) and 2.5 mg kg^−1^ (Cu); for class II 0.7–3.3 mg kg^−1^ (Zn) and 0.9–2.5 mg kg^−1^ (Cu); for class III < 0.7 mg kg^−1^ (Zn) and < 0.9 mg kg^−1^ (Cu). Among 27 investigated samples of agricultural use, most belong to class I (24 soils for zinc and 16 for copper) while the remaining to class II.

In Poland, according to Regulation [30], soils are divided into four categories of land use. For each group, the earth’s surface contamination is assessed based on the total metal contents. In term of soil use, samples under study were found in group I (residential, commercial, services, other built-up and green areas—33 samples), group II (agricultural areas, allotment gardens—28 samples), group III (wastelands, forests, green areas being subject to nature protection regulations—10 samples), and group IV (industrial and transportation areas—7 samples), Table S1. The metal contents (intervention values) for a specific soil use were exceeded for Pb, Zn, Cd for soil no. 4 (the total contents of analytes 193 mg kg^−1^, 760 mg kg^−1^ and 2.5 mg kg^−1^, respectively) and no. 11 (258 mg kg^−1^, 425 mg kg^−1^ and 2.5 mg kg^−1^, respectively). In order to assess the level of soil pollution and the environmental risk of metal mobile fractions, contamination factors (CFs) were calculated (Table S3). To calculate CFs (see Section 3.2), background values for anthropogenic fractions of metals in Polish Lowlands were used [58]. For about one-third of the sites, CF exceeds the values of 3 (3 ≤ CF < 6 considerable or CF ≥ 6 very high contamination [8,59]). Most sites were contaminated with copper (ca. 29% of all samples, including 9% with CF > 6). Soils collected in nine parks account for 40% of all samples with a high level of copper mobile fractions. Other authors also point out the high content of metals in the contaminated soil of urban green areas and human exposure to a direct and indirect contact with such soil [7,14,60]. Samples with high Cu content were taken, also, from locations along railways and roads with high traffic (especially near intersections or sharp turns). CF > 3 was also found for lead (15 samples, including 5 in parks), zinc (13 samples), and cadmium (1 sample). In this case, high CF values for Pb, Zn, and Cd should be attributed mainly to traffic emissions [61].

**Figure 3:**
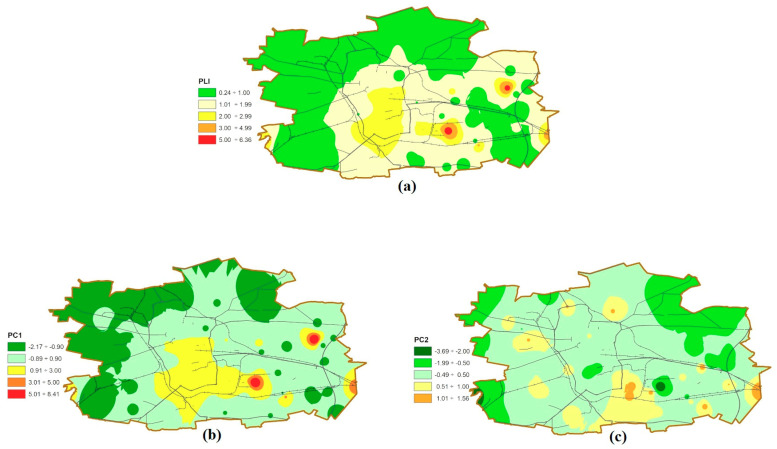
Spatial distribution of (**a**) pollution load index (PLI); (**b**) first principal component (PC1); (**c**) second principal component (PC2).

CF index makes it possible to assess the pollution of a particular site by individual metals. According to Wang [8], among indices of the soil pollution level, CF follows the most severe criteria. In order to estimate the overall soil contamination at a given location, pollution load index (PLI) was used (see Section 3.2). Contaminated soil (PLI > 1 [59]) was found in 30 sites (ca. 40% of all samples), Table S3. Figure 3a presents spatial distribution of PLI. Sampling sites identified as contaminated coincide with locations where CFs > 3 were found for at least one metal. The limit value was additionally slightly exceeded (PLI 1.1–1.4) for four samples collected near roads with average traffic density.

### 2.2. Multivariate Analysis and Spatial Distribution of the Principal Components (PCs)

**Figure 4:**
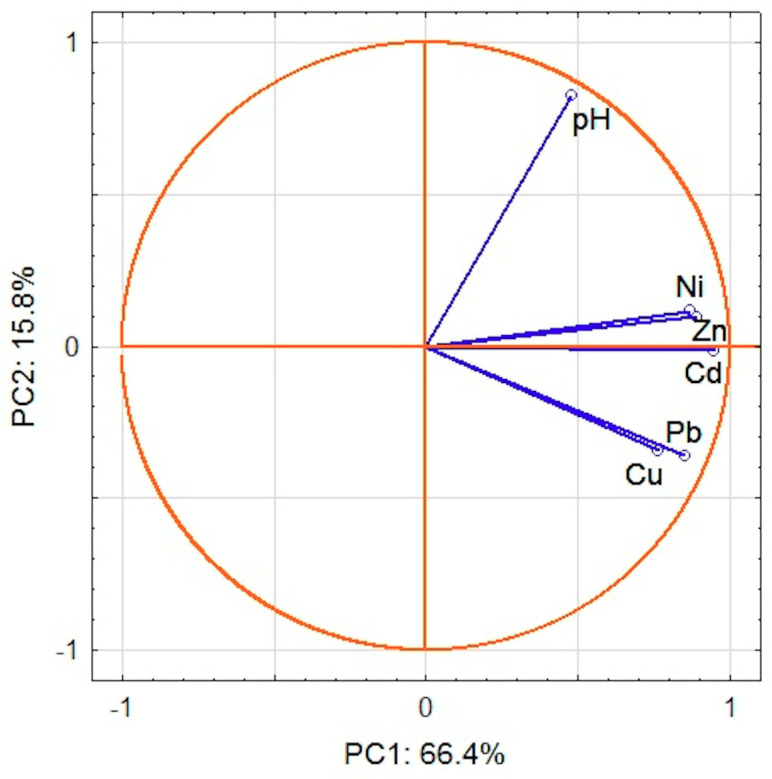
Loading plot of two main components PC1 and PC2.

**Figure 5:**
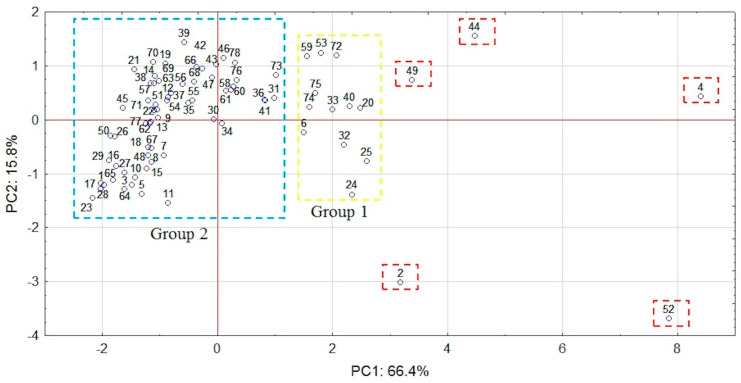
Score plot of PC1 versus PC2 for sampling points.

Following PCA, two main components (first principal component (PC1), second principal component (PC2)), which together account for over 80% of the data variability, were extracted. PC1 includes all investigated metals. Their factor loadings are within the range from 0.95 for Cd to 0.76 for Cu, whereas pH is involved in PC2 (factor loading is 0.82). PC2 has also weak loadings for Cu and Pb (factor loadings –0.34 and –0.36, respectively) and PC1 for pH (factor loading 0.48). The graphic representation of the main components is presented in Figure 4, which depicts the relations between variables in two-dimensional space. The score plot (Figure 5) shows that sampling points could be generally divided into two main groups (1 and 2). Five outliers (2,4,44,49,52) with the highest positive PC1 factor values can be identified. Sites 2 and 52 have also the lowest and site 44 the highest PC2 values. All outliers represent highly contaminated hot-spots (Figure 3b,c) of anthropogenic origin and are vital for the thorough characterization of investigated area. Following Abollino [62] on treatment of outliers in the field environmental studies, we decided to use complete dataset in PCA and CA calculations. At given locations, the content of at least two metals corresponds to considerable (CF 3-6) or high (CF > 6) contamination [59] further confirmed by high PLI values. In points 2, 44 and 49 PLI is within the range 3–5, while in sites 4 and 52 it exceeds 5. Group 1 consists of samples with highly positive PC1 values. With the exception of sites 6, 24, 25, and 32, PC2 values are positive. All samples are considerably or highly contaminated with lead and copper (a few also with zinc) and have PLI values in the range 2–3.

**Figure 6:**
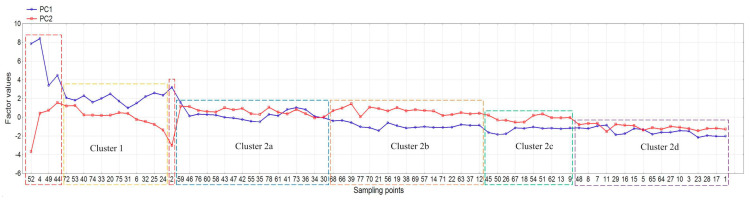
Factor values obtained from principal component analysis (PCA) for sampling points and classified according to the cluster analysis (CA) results.

CA results (Figure S2) are similar to the PCA score plot except that cluster 1 includes also all hot-spots. Unlike PCA score plot, in cluster 2 it is possible to distinguish particular sub-clusters (Figures S2–S4). According to the results of chemical analysis, CF and PLI indices together with factor values (PCA), sampling points were classified in four sub-clusters (2a,2b,2c,2d)—Figure 6 and Figure S3. In Table 2, the metal contents and pH values for clusters and five outliers were shown. Cluster 1 includes sampling points with highly positive PC1 factor values, the widest range of metal contents and soil pH. Most sites from cluster 1 are neutral, but acidic (no. 24) and slightly acidic (no. 6,25) points also occur. Soils from cluster 2 have significantly lower PC1 factor values and narrower scatter of metal concentrations. An important factor which divides cluster 2 into sub-clusters is pH. Most clusters, 2a and 2b, are characterized by neutral reaction. The remaining points are slightly acidic or alkaline. Cluster 2a contains soils with CF 1–3 (moderate contamination) and 3–6 (considerable contamination). 

Samples with CF > 3 are contaminated with copper or zinc. PLI in this cluster is 1–2, which indicates slight pollution of individual sites (four samples had PLI < 1). In turn, cluster 2b contains samples with moderate contamination (CF 2–3) and considering PLI, they are unpolluted. Cluster 2c gathers the soil samples characterized by highly negative PC1, low or moderate contamination in terms of contamination factor (CF), uncontaminated sites (pollution load index, PLI < 1) and lower pH reaction, which ranged from acidic to slightly acidic. Cluster 2d is characterized by the lowest negative PC1 and PC2 factor values and strongly acidic reaction. The values of CF and PLI are similar to cluster 2c, indicating no soil contamination.

As a result, groups of sites characterized by a specific pollution load were distinguished:

- Outliers (hot-spots)—no. 2 (energy willow crops fertilized with sludge from a wastewater treatment plant), no. 4, 44, 49, 52 (soils collected near roads with heavy traffic), no. 49 (located near the cement factory);
- Cluster 1—soils contaminated with Pb, Cu (and Zn) sampled within an area bounded by peripheral route railways; it is the oldest part of Łódź with compact buildings, still with tenements, narrow streets with heavy traffic (cars, buses, trams). Therefore, the main sources of pollution are transport, emissions from coal-fired furnaces, corrosion of building materials, metal construction elements, and service activities (workshops, commerce). Corrosion of building materials increases the pH (Figure 2), which in turn promotes metal accumulation. The source of metals is also storm water runoff from roofs [63];
- Cluster 2a—includes locations in the city center and its south and southeast parts (parks, grasslands, the oldest residential, industrial, service, and commercial areas). Half of these soil samples are contaminated with copper from sources other than sites for cluster 1 (e.g., fertilizers and copper-based pesticides);
- Cluster 2b—sites with low metal contents, situated outside the peripheral route railways; this area is dominated by housing estates and land used for agricultural purposes;
- Clusters 2c and 2d—unpolluted soils located on the outskirts of the city, differing in reaction: cluster 2c contains slightly acidic and acidic soils, belonging to groups I and II of soil use; cluster 2d gathers very acidic agricultural soils.

Following the rigorous statistical approach, outliers should be identified and eliminated prior to implementation of PCA, which is sensitive to outliers [64]. Generally, observation that differs from the majority of data is considered an outlier [65]. However, identification of outliers within real data is not effortless [66]. In the case of environmental data, anomalous results may be caused by an analytical error, but they can also indicate hot-spots [62]. Therefore, five outliers (2, 4, 44, 49, and 52) were eliminated, PCA was repeated and the results were compared with calculations on the complete dataset. This approach yielded two main components (Figure S4) with the contribution 79.6% to the total variance. PC1 was influenced by all metal contents. Their factor loadings were within the range from –0.95 to –0.79 for Cd and Cu, respectively. PC1 and PC2 were negatively correlated with pH (factor loadings –0.62 and –0.72, respectively). PC2 has also weak positive loadings for Pb (0.36) and Cu (0.29). Overall, the obtained statistical pattern is similar to the results of the analysis with outliers. Significant correlations are still observed among metals. The score plot (Figure S5) indicates that sampling points are divided into three main groups. Group I contains the same sites as selected by the first PCA and these sites are strongly influenced by PC1. The remaining points are in groups II and III. Elimination of hot-spots prompted diversity of pollution loads, especially in areas characterized by high metal contents like the center of Łódź. Therefore, the removal of hot-spots led to the loss of the data critical to the habitat and health of the inhabitants.

The picture that emerges from outliers’ elimination excludes already identified and highly polluted spots and highlights moderate polluted areas. Therefore, it overestimates uniformity of pollution distribution and disregards existing anthropogenic point sources of contamination.

## 3. Materials and Methods

### 3.1. Study Area and Soil Sampling

The city of Łódź, the capital of the District of Łódź (Łódzkie Voivodship), is located in central Poland (between latitudes 51°41′11”–51°51′40” N and longitudes 19°20′41”–19°38′30” E) [67]. Łódź is the third biggest city in Poland in terms of population (693,797 habitants in 2017) [68] and covers an area of approximately 293 km^2^ [69]. The central location of the city plays an important role in interregional and international transportation as one of the major communication hubs in Poland. Among the transportation facilities in Łódź, the most important are airport, railway, motorway A1 (north to south direction), 14, 72, and 91 national roads (primary routes). Over 50% of the city consists of built-up and urbanized land, 39.8% is agriculture land and 8.5% is forest land [69]. Łódź belongs to the transient zone between Southern Polish Highlands and Central Polish Lowlands and is situated at the borderline of two mezo-regions: Łask Plateau and Łódź Hills [70,71]. The highest point of the study area is located in the northeastern part of city at 284.1 m a.s.l. and gradually decreases towards the southwest to the elevation 163.6 m a.s.l. [67,72]. Łódź has a temperate climate and is affected by both oceanic and continental masses [71]. The annual average temperature in the period 1931–2017 ranged between 6 °C (1956) and 9.8 °C (2015). In 2017, the highest and the lowest monthly temperatures occur in August (19.1 °C) and January (−4.6 °C), respectively. In the years 1931–2017, the annual total precipitation ranged from 364 mm (1959) to 833 mm (2017). Atmospheric precipitation is usually higher in the spring–summer period than in the autumn–winter period (568 mm from May to October and 265 mm from November to April, 2017) [67,72,73]. The maximum annual load of metals delivered with precipitation to the study area was approximately: 0.16 mg Zn, 0.01 mg Cu, 0.002 mg Pb, 0.001 mg Ni, and 0.0003 mg Cd per kg of soil (2017) [69]. The annual prevailing wind is from the western, southwestern, and eastern (especially eastern (E) and southeastern (SE)) directions, which occurs at a frequency of 20%, 15%, and 14%, respectively. The frequency of the wind from northern and north-eastern directions is 8% and 5%, respectively [73,74]. It has been observed that the average wind speed is lower than 4 m/s in the city center, which is characterized by tall buildings. Additionally, in winter, the wind velocities decrease by up to 50%, which inhibit ventilation and intensify the deposition of particulate matter and accumulation of metals in the soils [12,72,75]. The highest pollution by particulate matter is usually observed in the city center. In 1985, the average annual concentration was 128 μg/m^3^ and decreased to 59 μg/m^3^ in 2000 [73]. Excessive air pollution in the city center was an effect of power plants equipped with insufficient fly ash removal installations. In subsequent years, dust emission from plants burdensome for air decreased from 4.0 t/km^2^ (2000) to 0.7 t/km^2^ (2013). Currently, the main source of particulate pollutants is surface emission related to individual heating systems of residential areas. In 2017, it occurred from 23 days (on the outskirts) to 82 days (in the city center), when permissible limits of particulate matter less than 10μm (PM 10) concentrations were exceeded [67,69,74,76].

In our study, 78 soil samples were collected in Łódź from sites characterized by various types of land use e.g., cropland, wasteland, urban area, industrial area (Table S1, Figure 1). Sampling sites were chosen randomly. Soil was collected using the Egner’s stick from the upper 20 cm surface layer (the depth of rooting for most plants). At each of the sites, one composite sample (approximately 0.5 kg of soil), consisting of 15–20 sub-samples, was taken. Soils were dried at room temperature for 1–2 weeks. Air-dried soils were passed through a 2-mm stainless steel sieve to remove debris [77]. Samples were stored in Ziploc bags.

Basic geographic data was acquired from the Head Office of Land Surveying and Cartography [78]. The location of sampling sites was determined by handheld Global Positioning System (GPS) (MobileMapper 50). The inverse distance weighted (IDW) [79] of ArcMap 9.2 (Esri Redlands, Ca, USA) software was used to interpolate all study parameters and generate geochemical maps [49].

### 3.2. Analytical and Statistical Methods

The organic matter (OM) content was determined (Table S1) by weight loss after ignition at 550 °C in a muffle furnace [80]. For the analysis of particle size composition, the areometric-sieve method [81] was applied. Soil pH was measured by the potentiometric method in 1 mol L^−1^ KCl solution (soil:KCl ratio 1:2.5 *m*/*V*) [82]. In order to determine contents of metal mobile fractions (Cu, Zn, Pb, Cd, Ni), samples were subjected to extraction with 1 mol L^−1^ HCl solution (soil:HCl ratio 1:5 m/V) [45,46]. Total metal contents (Table S3) were determined after microwave digestion with an Anton Paar Multiwave 3000 (Graz, Austria) closed system instrument. Approximately 0.5 g of soil sample was digested with aqua regia (HNO_3_:HCl ratio 2:6 V/V) and concentrated HF (3 mL) in the PTFE vessel. Mineralization was carried out for 30 min at 240 °C under pressure of 6 bar. The vessel was allowed to cool, 9 mL of 4% H_3_BO_3_ was added and digestion was continued for 15 min. Mobile fractions and total metal concentrations were determined by flame atomic absorption spectrometry (FAAS) with the GBC 932 plus spectrometer (Melbourne, Australia).

During soil collection, samples preparation and every analytical step, special precautions were taken to reduce contamination of samples. All laboratory glassware was cleaned by immersion in 10% HNO_3_ (for minimum 24 h) and rinsed thoroughly with deionized water. Each reagent was analytical or Suprapur quality. For preparation of calibration curves, standard solutions of metals (1000 mg/L) purchased from Merck were used. All other reagents were obtained from POCh (Poland). Deionized water with electrical conductivity 0.05 μS was used (water deionizer system Polwater, Cracow, Poland). Dilute HCl was added to each calibration standard to obtain the same acidic matrix. For each analytical procedure, a blank experiment was carried out. All measurements were performed in triplicate on separate soil samples. Certified reference material (CRM) for extraction with 1 mol L^−1^ HCl was not commercially available. For this reason, CRM 7002 Light Sandy Soil obtained from Analytika Co. Ltd. Czech Republic was analyzed to provide quality assurance. Metal contents were determined after extraction by cold 2 mol L^−1^ HNO_3_. The recoveries ranged between 93% for Ni and 110% for Cd. The analytical precision was assessed by replicated soil samples (seven parallel aliquots). The relative standard deviations (RSD) were calculated and ranged from 3% to 7%.

Basic statistics, normality test, principal component analysis (PCA), and cluster analysis (CA) were performed with the Statistica 10. The distribution normality of the data was examined by the Kolmogorov–Smirnov (K–S) test. Column auto scaling was performed before the implementation of PCA and CA. CA was developed using Ward’s method and the squared Euclidean distance was applied to calculate the cluster distance. Additionally, variability of metal concentrations was assessed by coefficient of variation (CV) [52]. Statistical methods were supported by the following geochemical parameters: contamination factor (CF) [59] and pollution load index (PLI) [83], which were calculated using the following equations:

CF = C_m_/B_m_ (1)

where: C_m_ is the measured metal content, B_m_ is the geochemical background of the metal;

PLI = (CF_1_ · CF_2_ ·…· CF_n_)^1/n^ (2)

where: n is the number of analyzed metals, CF is the contamination factor for each metal.

## 4. Conclusions

In this work, the contents of mobile fractions of Cd, Cu, Ni, Pb, and Zn in urban soils of Łódź were determined. The spatial distribution of metals and the PLI values show that the highest metal concentrations occur in the city center and in its southern and southeastern part. This area, covering the oldest residential, commercial, and industrial districts, is influenced by contamination from many point sources. Heavy metal concentrations remain even after their source has been removed, and therefore, their content is associated with either historical or current pollution. After the crisis of the 1980s and 1990s, the structure of land use changed, but a large load of metal contamination is still observed in this area. The prevailing wind direction is another factor affecting the distribution of mobile metal fractions. Although in the western and northwestern part of the city there are numerous point sources of industrial and traffic emissions, this area is characterized by a low content of metal mobile fractions. In Łódź, the wind from the west dominates and pollutants are carried towards the city center. In turn, the compact buildings and narrow streets of the downtown change the wind directions. This causes metals accumulation in unpredictable ways. Corrosion of building materials also affects metal concentration and gives rise to a higher soil pH. Finally, runoff of rainwater from the roofs of buildings is a neglected, but important, source of contamination of urbanized areas. The distribution of mobile metal fractions also coincides with the order of suburban agricultural land incorporation in the administrative boundaries of Łódź and the change of their use. The highest metal concentrations occur in the oldest part of the city. The lowest contents were observed on the outskirts, i.e., agricultural areas incorporated within the city boundaries in the years 1945–2000.

Soil is subjected to an increasing impact of anthropopressure, which is particularly severe in urban areas. Monitoring of urban soil pollution is an essential element of actions aimed to improve the quality of life for the city residents. Particular attention should be given to the problems of pollution in gardens, parks and other recreational areas due to the health risk as a result of the direct contact of habitants (and especially children) with contaminated soil. Another group exposed to the prolonged contact with pollution from public and private transport is the inhabitants of buildings located near streets and roads subjected to heavy traffic. According to the European Environment Agency, soil contamination concerns almost 250,000 sites in Europe [28]. Therefore, methods for creating comprehensive information package, which may support soil remediation, appropriate spatial development, and increasing public awareness of prevention of environmental pollution, should be developed. The combined approach based on PCA, CA, PLI, and GIS is invaluable in this respect. It helps to identify hot-spots requiring clean-up and visualizes overall contamination of urban soils.

## Figures and Tables

**Table 1:** Summary of descriptive statistics for metal contents (mg kg^−1^) and pH in examined soil samples (n = 78).

Header	Pb	Cd	Cu	Ni	Zn	pH
Mean	21.6	0.34	8.39	2.10	42.8	6.0
Minimum	4.05	0.05	0.77	0.33	2.45	3.5
Q_1_	10.4	0.15	3.10	0.90	9.58	5.0
Median	15.2	0.24	5.20	1.48	16.7	6.4
Q_3_	23.6	0.42	11.6	2.96	60.9	7.0
Maximum	163	2.09	48.4	7.63	358	8.0
Skewness	4.39	3.13	2.45	1.43	2.89	−0.59
Kurtosis	24.5	12.9	6.91	1.81	10.4	−1.01
K–S p	<0.01	<0.01	<0.01	<0.05	<0.01	<0.05
CV [%]	101	94.1	108	76.5	140	20.7

Q_1_—lower quartile; Q_3_—upper quartile; K–S—Kolmogorov–Smirnov; CV—coefficient of variation.

**Table 2:** Metal contents (mg kg^−1^) and pH values for clusters and outliers.

	Pb	Cd	Cu	Ni	Zn	pH
Cluster 1	20.0–52.3	0.36–0.74	6.13–33.4	2.43–4.83	47.3–208	5.2–7.2
mean	**37.2**	**0.55**	**17.5**	**3.46**	**102**	**6.8**
Cluster 2a	10.4–27.4	0.19–0.49	4.09–18.4	1.06–5.92	12.0–91.6	6.0–7.4
mean	**18.3**	**0.34**	**9.19**	**2.56**	**42.6**	**6.8**
Cluster 2b	6.53–22.7	0.11–0.28	1.14–7.62	0.53–2.00	3.84–21.2	6.1–7.6
mean	**13.0**	**0.19**	**4.34**	**1.12**	**13.5**	**6.7**
Cluster 2c	4.05–16.8	0.08–0.25	0.77–3.97	0.43–1.42	3.07–20.3	5.0–6.0
mean	**11.6**	**0.16**	**2.61**	**1.01**	**10.4**	**5.4**
Cluster 2d	7.89–24.9	0.05–0.24	1.09–8.11	0.33–3.60	2.45–23.1	3.5–4.6
mean	**12.8**	**0.16**	**3.11**	**1.14**	**8.51**	**4.1**
Site 52	163	1.29	41.8	6.52	204	5.2
Site 4	103	2.09	16.4	6.26	358	8.0
Site 49	34.4	1.01	16.3	6.09	105	7.3
Site 44	20.4	1.32	12.6	7.63	183	7.6
Site 2	48.6	0.76	48.4	3.89	65.2	4.3

## Supplementary Materials

The following are available online, Figure S1: Comparison of the lead and zinc contents for sampling points, which are characterized by higher lead contents, Figure S2. Dendrogram for sampling sites, Figure S3. Dendrogram for sampling sites with scale of the linkage distance reducing to 50, Figure S4. Loading plot of the two main components PC1 and PC2 for PCA without outliers, Figure S5. Score plot of PC1 versus PC2 for PCA without outliers Table S1: Characteristic of sampling sites, Table S2. Metal mobile fraction (M) and total (T) contents in soils. Table S3. Contamination factors (CF) and pollution load index (PLI)

## References

[1] Nazzal Y.H., Al-Arifi N.S.N., Jafri M.K., Kishawy H.A., Ghrefat H., El-Waheidi M.M., Batayneh A., Zumlot T. (2015). Multivariate statistical analysis of urban soil contamination by heavy metals at selected industrial locations in the Greater Toronto area, Canada. Geol. Croat..

[2] Jin Y., O‘Connor D., Ok Y.S., Tsang D.C.W., Liu A., Hou D. (2019). Assessment of sources of heavy metals in soil and dust at children‘s playgrounds in Beijing using GIS and multivariate statistical analysis. Environ. Int..

[3] Barbieri M. (2016). The Importance of Enrichment Factor (EF) and Geoaccumulation Index (Igeo) to Evaluate the Soil Contamination. J. Geol. Geophys..

[4] Nazarpour A., Watts M.J., Madhani A., Elahi S. (2019). Source, Spatial Distribution and Pollution Assessment of Pb, Zn, Cu, and Pb Isotopes in urban soils of Ahvaz City, a semi-arid metropolis in southwest Iran. Sci. Rep..

[5] Techogenic Soil—Soil of the Year 2020. http://ptg.sggw.pl/en/gleba-technogeniczna-gleba-roku-2020/.

[6] Ali M.H., Mustafa A.R.A., El-Sheikh A.A. (2016). Geochemistry and spatial distribution of selected heavy metals in surface soil of Sohag, Egypt: A multivariate statistical and GIS approach. Environ. Earth Sci..

[7] Oka G.A., Thomas L., Lavkulich L.M. (2014). Soil assessment for urban agriculture: A Vancouver case study. J. Soil Sci. Plant Nut..

[8] Wang C., Ye Z., Wang W., Jin M. (2016). Traffic-Related Heavy Metal Contamination in Urban Areas and Correlation with Traffic Activity in China. Transp. Res. Rec. J. Transp. Res. Board.

[9] Han X., Lu X., Qinggeletu, Wu Y. (2017). Health Risks and Contamination Levels of Heavy Metals in Dusts from Parks and Squares of an Industrial City in Semi-Arid Area of China. Int. J. Environ. Res. Public Health.

[10] Zhang J., Hua P., Krebs P. (2017). Influences of land use and antecedent dry-weather period on pollution level and ecological risk of heavy metals in road-deposited sediment. Environ. Pollut..

[11] Sakan S.M., Đordević D.S., Manojlović D.D. (2010). Trace elements as tracers of environmental pollution in the canal sediments (alluvial formation of the Danube River, Serbia). Environ. Monit. Assess..

[12] Wang X.S., Qin Y., Sang S.X. (2005). Accumulation and sources of heavy metals in urban topsoils: A case study from the city of Xuzhou, China. Environ. Geol..

[13] Tytła M. (2020). Identification of the chemical forms of heavy metals in municipal sewage sludge as a critical element of ecological risk assessment in terms of its agricultural or natural use. Int. J. Environ. Res. Public Heatlh.

[14] Horváth A., Kalicz P., Farsang A., Balázs P., Berki I., Bidló A. (2018). Influence of human impacts on trace metal contamination in soils of two Hungarian cities. Sci. Total Environ..

[15] Senila M., Levei E.A., Senila L.R. (2012). Assessment of metals bioavailability to vegetables under field conditions using DGT, single extractions and multivariate statistics. Chem. Cent. J..

[16] Xu H., Wang Y., Liu R., Wang M., Zhang Y. (2019). Spatial Distribution, Chemical Speciation and Health Risk of Heavy Metals from Settled Dust in Qingdai Urban Area. Atmosphere.

[17] Chen X., Lu X., Yang G. (2012). Sources identification of heavy metals in urban topsoil from inside the Xi‘an Second Ringroad, NE China using multivariate statistical methods. Catena.

[18] Simon E., Vidic A., Braun M., Fábián I., Tóthmérész B. (2013). Trace element concentrations in soils along urbanization gradients in the city of Wien, Austria. Environ. Sci. Pollut. Res. Int..

[19] Ferri R., Hashim D., Smith D.R., Guazzetti S., Donna F., Ferretti E., Curatolo M., Moneta C., Beone G.M., Lucchini R.G. (2015). Metal contamination of home garden soils and cultivated vegetable in the province of Breschia, Italy: Implications for human exposure. Sci. Total Environ..

[20] Charzyński P., Hulisz P., Bednarek R., Piernik A., Winkler M., Chmurzyński M. (2015). Edifisols – a new soil unit of technogenic soils. J. Soil. Sediment..

[21] Dąbkowska-Naskręt H., Różański S., Bartkowiak A. (2016). Forms and mobility of trace elements in soils of park areas from the city of Bydgoszcz, north Poland. Soil Sci. Ann..

[22] Rahmonov O., Banaszek J., Pukowiec-Kurda K. (2019). Relationship Between Heavy Metal Concentration in Japanese Knotweed (*Reynoutria Japonica* Houtt.) Tissues and Soil in Urban Parks in Southern Poland. IOP Conf. Ser. Earth Environ. Sci..

[23] Różański S.Ł., Kwasowski W., Peñas Castejón J.M., Hardy A. (2018). Heavy metal content and mobility in urban soils of public playgrounds and sport facility areas, Poland. Chemosphere.

[24] Zwolak A., Sarzyńska M., Szpyrka E., Stawarczyk K. (2019). Sources of Soil Pollution by Heavy Metals and Their Accumulation in Vegetables: A Review. Water Air Soil Pollut..

[25] Bielińska E.J., Mocek-Płóciniak A. (2010). Impact on Ecochemical Soil Conditions on Selected Heavy Metals Content in Garden Allotment Vegetables. Pol. J. Environ. Stud..

[26] Kabała C., Chodak T., Szerszeń L., Karczewska A., Szopka K., Frątczak K. (2009). Factors influencing the concentration of heavy metals in soils of allotment gardens in the city of Wrocław, Poland. Fresenius Environ. Bull..

[27] Fritsch C., Giraudoux P., Coeurdassier M., Douay F., Raoul F., Pruvot C., Waterlot C., de Vaufleury A., Scheifler R. (2010). Spatial distribution of metals in smelter-impacted soils of woody habitats: Influence of landscape and soil properties, and risk for wildlife. Chemosphere.

[28] European Environment Agency Topics. Soil. Contamination from Local Sources. https://www.eea.europa.eu/themes/soil/soil-threats.

[29] IUSS Working Group WRB (2015). World Reference Base for Soil Resources 2014, Update 2015 International Soil Classification System for Naming Soils and Creating Legends for Soil Maps. World Soil Resources Reports No. 106. FAO, Rome. http://www.fao.org/3/i3794en/I3794en.pdf.

[30] Minister of the Environment (2016). Regulation of the Minister of the Environment of 1 September 2016 on the Method of the Contamination Assessment of the Earth Surface. Journal of Laws of 2016.

[31] Lamprecht M., Kobojek E., Habrel M. (2013). Origins and Spatial Development of Łódź. Lviv and Łódź at the Turn of 20th Century. Historical Outline and Natural Environment.

[32] Marcińczak S. (2012). The evolution of spatial patterns of residential segregation in Central European Cities: The Łódź Functional Urban Region from mature socialism to mature post-socialism. Cities.

[33] Wójcik M. (2016). Selected problems of contemporary socio-spatial changes in peri-urban areas of the city of Łódź (Poland). Geogr. Pol..

[34] Wójcik M., Tobiasz-Lis P., Dmochowska-Dudek P. (2018). Uneven Development of a Post-Industrial City as Exemplified by Łódź (Poland). Mitteilungen der Österreichischen Geographischen Gesellschaft.

[35] Czarnowska K., Walczak J. (1988). Distribution of zinc, lead and manganese in soils of Łódź City. Rocz. Glebozn..

[36] Czarnowska K. (1992). Distribution of copper, chromium, nickel, cobalt and cadmium in soils of the city of Łódź. Rocz. Glebozn..

[37] Jankiewicz B., Ptaszyński B., Turek A. (1999). Spectrophotometric Determination of Copper(II) in Samples of Soil from Selected Allotment Gardens in Lodz. Pol. J. Environ. Stud..

[38] Jankiewicz B., Ptaszyński B., Wieczorek M. (2000). Spectrophotometric Determination of Cadmium(II) in Soil of Allotment Gardens in Łódź. Pol. J. Environ. Stud..

[39] Jankiewicz B., Ptaszyński B., Wieczorek M. (2001). Spectrophotometric Determination of Lead in the Soil of Allotment Gardens in Łódź. Pol. J. Environ. Stud..

[40] Jankiewicz B., Adamczyk D. (2007). Assessing Heavy Metal Content in Soils Surrounding the Łódź EC4 Power Plant, Poland. Pol. J. Environ. Stud..

[41] Jankiewicz B., Adamczyk D. (2010). Assessing Heavy Metal Content in Soils Surrounding a Power Plant. Pol. J. Environ. Stud..

[42] Szynkowska M.I., Pawlaczyk A., Leśniewska E., Paryjczak T. (2009). Toxic Metal Distribution in Rural and Urban Soil Samples Affected by Industry and Traffic. Pol. J. Environ. Stud..

[43] Polish Committee for Standardization (2001). Polish Standard PN-ISO 11259 (2001). Soil Quality—Simplified Soil Description.

[44] Kwon M.J., Boyanov M.I., Yang J.S., Lee S., Hwang Y.H., Lee J.Y., Mishra B., Kemner K.M. (2017). Transformation of zinc-concentrate in surface and subsurface environments: Implications for assessing zinc mobility/toxicity and choosing an optimal remediation strategy. Environ. Pollut..

[45] Polish Committee for Standardization (1992). Polish Standard. PN-R-04016:1992. Agrochemical Soil Analyses—Determination of Assimilated Zinc Contents.

[46] Polish Committee for Standardization (1992). Polish Standard. PN-R-04017:1992. Agrochemical Soil Analyses—Determination of Assimilated Copper Contents.

[47] Senila M. (2014). Real and simulated bioavailability of lead in contaminated and uncontaminated soils. J. Environ. Health Sci. Eng..

[48] Radecki-Pawlik A., Wałęga A., Młyński D., Młocek W., Kokoszka R., Tokarczyk T., Szalińska W. (2020). Seasonality of mean flows as a potential tool for the assessment of ecological processes: Mountain rivers, Polish Carpathians. Sci. Total Environ..

[49] Kelepertzis E. (2014). Accumulation of heavy metals in agricultural soils of Mediterranean: Insights from Argolida basin, Peloponnese, Greece. Geoderma.

[50] Gulan L., Milenkovic B., Zeremski T., Milic G., Vuckovic B. (2017). Persistent organic pollutants, heavy metals and radioactivity in the urban soil of Priština City, Kosovo and Metohija. Chemosphere.

[51] Ma L., Wang L., Jia Y., Yang Z. (2016). Arsenic speciation in locally grown rice grains from Hunan Province, China: Spatial distribution and potential health risk. Sci. Total Environ..

[52] Karim Z., Qureschi B.A., Mumtaz M., Qureschi S. (2014). Heavy metal content in urban soils as an indicator of anthropogenic and natural influences on landscape of Karachi—A multivariate spatio-temporal analysis. Ecol. Indic..

[53] Draszawka-Bolzan B. (2017). Effect of pH and soil environment. WNOFNS.

[54] Mazurek R., Kowalska J., Gąsiorek M., Zadrożny P., Józefowska A., Zaleski T., Kępka W., Tymczuk M., Orłowska K. (2017). Assessment of heavy metals contamination in surface layers of Roztocze National Park forest soils (SE Poland) by indices of pollution. Chemosphere.

[55] Hou D., O’Connor D., Nathanail P., Tian L., Ma Y. (2017). Integrated GIS and multivariate statistical analysis for regional scale assessment of heavy metal soil contamination: A critical review. Environ. Pollut..

[56] Ander E.L., Johnson C.C., Cave M.R., Palumbo-Roe B., Nathanail C.P., Lark R.M. (2013). Methodology for the determination of contaminants in English soil. Sci. Total Environ..

[57] Jadczyszyn J., Niedźwiecki J., Debaene G. (2016). Analysis of Agronomic Categories in Different Soil Texture Classification Systems. Pol. J. Soil Sci..

[58] Pasieczna A. (2003). Atlas of Urban Soils Contamination in Poland.

[59] Elnazer A.A., Salman S.A., Saleem E.M., Abu El Ella E.M. (2015). Assessment of Heavy Metals Pollution and Bioavailability in Roadside Soil of Alexandria-Marsa Matruh Highway, Egypt. Int. J. Ecol..

[60] Setälä H., Francini G., Allen J.A., Jumpponen A., Hui N., Kotze D.J. (2017). Urban parks provide ecosystem services by retaining metals and nutrients in soils. Environ. Pollut..

[61] Świetlik R., Strzelecka M., Trojanowska M. (2013). Evaluation of Traffic-Related Heavy Metals Emissions Using Noise Barrier Road Dust Analysis. Pol. J. Environ. Stud..

[62] Abollino O., Malandrino M., Giacomino A., Mentasi E. (2011). The role of chemometrics in single and sequential extraction assays: A review: Part I. Extraction procedures, uni- and bivariate techniques and multivariate variable reduction techniques for pattern recognition. Anal. Chim. Acta.

[63] Sakson G. (2017). Efficiency of heavy metals removal during roof runoff infiltration through vegetated soil. Environ. Prot. Eng..

[64] Serneels S., Verdonck T. (2008). Principal component analysis for data containing outliers and missing elements. Comput. Stat. Data Anal..

[65] Chen X., Zhang B., Wang T., Bonni A., Zhao G. (2020). Robust principal component analysis for accurate outlier sample detection in RNA-Seq data. BMC Bioinform..

[66] Rousseeuw P.J., Hubert M. (2018). Anomaly detection by robust statistics. WIREs Data Mining Knowl. Discov..

[67] Statistical Office in Łódź Data on Łódź. Geography, Meteorology. Geographic Location of the City. https://lodz.stat.gov.pl/en/information-about-voivodship/capital-of-voivodship-614/data-on-lodz-2018/.

[68] Statistical Office in Łódź Data on Łódź. Population. Population Based on Balances by Former Office Agencies of the City of Łódź Office (2010, 2015, 2016, 2017). https://lodz.stat.gov.pl/en/information-about-voivodship/capital-of-voivodship-614/population-data-on-lodz-2018/.

[69] Voivodeship Inspectorate for Environmental Protection Report on the State of Environment in the Łódzkie Voivodship Studied in 2017. https://www.wios.lodz.pl/Publikacje_WIOS,12.

[70] Kondracki J. (2009). Regional Geography of Poland.

[71] Kobojek E., Pielesiak I., Kobojek E., Habrel M. (2013). Łódź in Geographical Space. Lviv and Łódź at the Turn of 20th Century. Historical Outline and Natural Environment.

[72] Kobojek E., Kobojek E., Habrel M. (2013). Environmental Determinants of Development and Physiography of Łódź. Lviv and Łódź at the Turn of 20th Century. Historical Outline and Natural Environment.

[73] Łódź Land Information System The Łódź Atlas. Climate. http://www.mapa.lodz.pl/index.php?strona=atlas.

[74] Bartnik A., Marcinkowski M. (2015). Spatial variability of precipitation in the area of Lodz. Acta Universitatis Lodziensis. Folia Geographica Physica.

[75] Kosheleva N.E., Vlasov D.V., Korlyakov I.D., Kasimov N.S. (2018). Contamination of urban soils with heavy metals in Moscow as affected by building development. Sci. Total Environ..

[76] Bem H., Gallorini M., Rizzio E., Krzemińska M. (2003). Comparative studies on the concentrations of some elements in the urban air particulate matter in Lodz City of Poland and in Milan, Italy. Environ. Int..

[77] Polish Committee for Standardization (1997). Polish Standard. PN-R-04031:1997. Agrochemical Soil Analyses—Sampling.

[78] Head Office of Geodesy and Cartography. www.gugik.gov.pl.

[79] Xie Y.F., Chen T.B., Lei M., Yang J., Guo Q.J., Song B., Zhou X.Y. (2011). Spatial distribution of soil heavy metal pollution estimated by different interpolation methods: Accuracy and uncertainty analysis. Chemosphere.

[80] Caravaca F., Lozano Z., Rodriguez-Caballero G., Roldán A. (2017). Spatial shifts in soil microbial activity and degradation of pasture civer caused by prolonged exposure to cement dust. Land Degrad. Dev..

[81] Polish Committee for Standardization (1998). Polish Standard (1998). PN-R-04032:1998. Soils and Mineral Grounds. Soil Sampling and Grain Size Distribution.

[82] Polish Committee for Standardization (1997). Polish Standard PN-ISO 10390. Soil Quality–Determination of pH.

[83] Pobi K.K., Satpati S., Dutta S., Nayek S., Saha R.N., Gupta S. (2019). Sources evaluation and ecological risk assessment of heavy metals accumulated within a natural stream of Durgapur industrial zone, India, by using multivariate analysis and pollution indices. Appl. Water Sci..

