# Supplementary material for: Comprehensive Evaluation of Metal Pollution in Urban Soils of a Post-Industrial City—A Case of Łódź, Poland
Source: Molecules. 2020 Sep 22;25(18):4350. doi: 10.3390/molecules25184350 (PMC7570559; doi:10.3390/molecules25184350)
Supplement: Supplementary file 1 [file molecules-25-04350-s001.pdf]

Table S1. Characteristic of sampling sites.

No	Textural classification [43]	pH-KCl	OM [%]	Type of land use	Group [30]	No	Textural classification [43]	pH-KCl	OM [%]	Type of land use	Group [30]
**1**	sand	3.6	4.4	cropland	II	**24**	sand	5.2	8.1	park	I
**2**	loamy sand	4.3	12.4	cropland	II	**25**	sand	6.5	9.3	park	I
**3**	sandy loam	4.0	4.4	wasteland	III	**26**	sand	5.1	1.8	cropland	II
**4**	sandy loam	8.0	6.7	cropland	II	**27**	loamy sand	4.1	3.8	cropland	II
**5**	sandy loam	3.9	4.3	cropland	II	**28**	loamy sand	3.8	3.6	cropland	II
**6**	loamy sand	6.3	9.9	wasteland	III	**29**	loamy sand	4.4	3.2	cropland	II
**7**	loamy sand	4.3	5.4	cropland	II	**30**	sand	6.5	7.3	park	I
**8**	sandy loam	4.6	3.6	cropland	II	**31**	loamy sand	7.0	7.2	park	I
**9**	sandy loam	5.7	5.1	cropland	II	**32**	sand	7.1	5.5	park	I
**10**	sandy loam	4.1	5.1	cropland	II	**33**	sand	7.1	4.7	urban and commercial area	I
**11**	sandy loam	3.9	4.0	cropland	II	**34**	sand	6.3	7.9	park	I
**12**	sand	6.4	5.0	cropland	II	**35**	sand	6.0	6.8	commercial area	I
**13**	sand	5.6	3.2	wasteland	III	**36**	sand	6.8	7.2	park	I
**14**	sand	6.6	3.7	wasteland	III	**37**	sand	6.3	4.4	lawn	I
**15**	sandy loam	4.4	3.3	cropland	II	**38**	sand	6.6	2.7	urban and service area	I
**16**	sandy loam	4.3	7.8	cropland	II	**39**	sand	7.6	1.9	industrial area	IV
**17**	sand	3.8	2.9	cropland	II	**40**	sand	7.0	6.0	urban area	I
**18**	sand	5.0	4.6	grassland	II	**41**	sand	7.2	5.0	traffic area	IV
**19**	sand	7.0	3.6	traffic area	IV	**42**	sand	7.0	3.8	park	I
**20**	sand	7.1	6.7	commercial and service area	I	**43**	sand	7.2	5.4	recreation area	I
**21**	sand	6.8	1.8	wasteland	III	**44**	sand	7.6	10.0	residential area	I
**22**	loamy sand	6.1	4.0	cropland	II	**45**	sand	5.6	2.7	residential area	I
**23**	sand	3.5	2.6	cropland	II	**46**	sand	7.0	5.7	residential area	I

Table S1. Characteristic of sampling sites (continued).

**No**	Textural classification [43]	pH-KCl	OM [%]	Type of land use	Group [30]	**No**	Textural classification [43]	pH-KCl	OM [%]	Type of land use	Group [30]
**47**	sand	6.9	4.8	grassland	II	**63**	loamy sand	6.5	4.0	wasteland	III
**48**	sand	4.4	3.4	traffic area	IV	**64**	sandy loam	4.0	4.1	wasteland	III
**49**	sand	7.3	4.6	wasteland	III	**65**	sandy loam	4.0	4.4	cropland	II
**50**	sand	5.0	4.3	cropland	II	**66**	sand	7.3	2.7	urban area	I
**51**	sand	6.0	6.5	residential area	I	**67**	sand	5.0	7.3	park	I
**52**	sand	5.2	6.9	industrial and commercial area	IV	**68**	sand	7.2	3.0	urban area	I
**53**	sand	7.1	7.5	industrial and commercial area	IV	**69**	sand	6.7	4.2	residential area	I
**54**	sand	5.8	5.5	cropland	II	**70**	sand	7.2	2.7	residential area	I
**55**	sand	6.0	9.4	grassland	II	**71**	sand	6.2	2.9	wasteland	III
**56**	loamy sand	6.6	4.8	urban and commercial area	I	**72**	sand	7.2	4.3	urban area	I
**57**	sand	6.6	3.2	wasteland	III	**73**	sand	7.4	5.9	urban area	I
**58**	sand	6.7	7.6	grassland	II	**74**	sand	6.6	9.5	park	I
**59**	loamy sand	7.3	6.5	traffic area	IV	**75**	sand	7.2	8.2	allotment gardens	II
**60**	sand	6.9	3.9	grassland	II	**76**	sand	7.0	4.9	recreation area	I
**61**	loamy sand	6.6	5.0	urban and commercial area	I	**77**	sand	6.2	3.6	residential area	I
**62**	loamy sand	5.6	6.3	urban and commercial area	I	**78**	sand	7.3	4.0	residential area	I

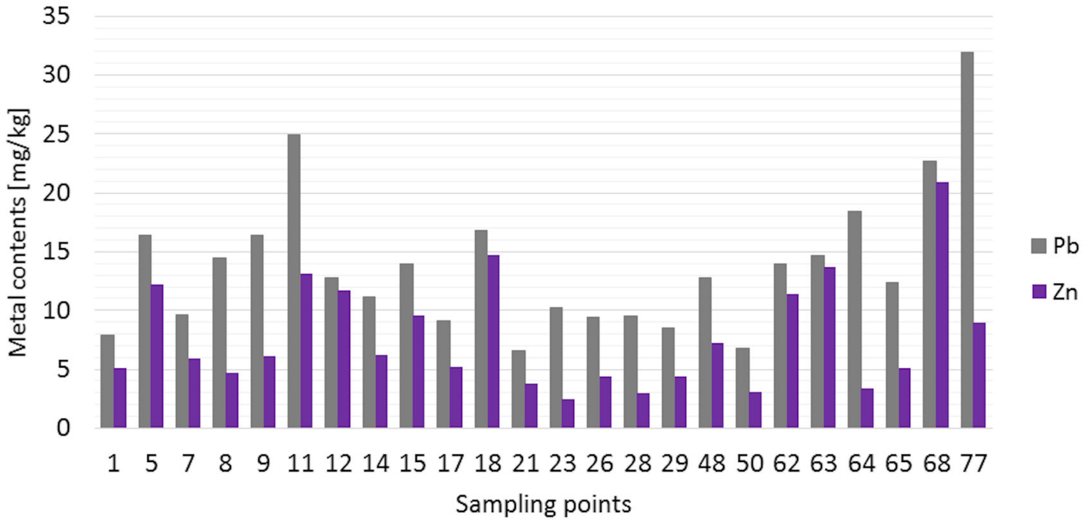

Figure S1. Comparison the lead and zinc contents for sampling points, which are characterized by higher lead contents.

Table S2. Metal mobile fraction (M) and total (T) contents in soils.

No.	Pb		Cd		Cu		Ni		Zn	
	M [mg·kg^{-1}]	T [mg·kg^{-1}]	M [mg·kg^{-1}]	T [mg·kg^{-1}]	M [mg·kg^{-1}]	T [mg·kg^{-1}]	M [mg·kg^{-1}]	T [mg·kg^{-1}]	M [mg·kg^{-1}]	T [mg·kg^{-1}]
1	7.89	12.8	0.14	0.92	1.09	2.53	0.57	6.80	5.09	11.9
2	48.6	61.9	0.76	1.55	48.4	68.9	3.89	14.4	65.2	144
3	15.9	22.7	0.21	0.87	3.49	5.84	0.65	11.3	16.2	41.4
4	103	193.4	2.09	2.54	16.4	45.9	6.26	23.2	357	760
5	16.4	25.1	0.22	0.54	6.09	9.01	0.97	14.0	12.2	38.7
6	33.0	44.7	0.62	0.79	17.4	33.8	3.00	29,1	66.9	172
7	9.58	18.6	0.17	1.04	2.44	3.76	3.60	23.9	5.94	21.7
8	14.5	22.4	0.19	0.77	2.45	3.58	1.95	13.1	4.66	25.0
9	16.4	23.2	0.22	0.64	3.04	4.11	0.90	11.3	6.11	27.3
10	14.3	24.3	0.22	0.63	2.99	5.24	0.98	12.9	14.7	58.1
11	24.9	258	0.23	2.46	8.11	39.2	1.66	38.6	13.1	425
12	12.8	25.4	0.24	0.93	5.48	9.74	1.11	12.2	11.7	39.6
13	13.4	22.5	0.19	63.6	3.36	6.10	0.79	11.9	13.5	40.2
14	11.2	17.6	0.17	0.58	3.15	5.82	1.10	11.3	6.23	23.7
15	14.0	22.3	0.24	0.91	4.25	5.79	1.37	15.0	9.58	45.4
16	8.73	18.5	0.11	1.24	2.82	9.33	0.81	10.4	9.39	51.9
17	9.13	17.4	0.08	1.17	1.54	2.99	0.61	7.54	5.23	27.5
18	16.8	22.3	0.17	0.40	3.65	7.36	1.16	14.6	147	48.4
19	8.93	15.1	0.14	0.21	3.21	4.52	1.65	11.7	13.7	25.0
20	35.8	270	0.57	7.25	22.7	53.6	4.83	24.6	83.4	152
21	6.53	12.7	0.13	0.17	1.14	2.28	0.63	9.66	3.84	14.4
22	12.1	17.7	0.18	1.01	4.32	6.83	1.01	11.1	15.0	56.4
23	10.2	21.9	0.05	0.96	2.35	4.94	0.33	11.7	2.45	35.2
24	45.0	62.2	0.74	0.87	24.5	34.4	4.19	23.2	67.9	107
25	41.5	58.8	0.63	1.30	32.4	48.4	3.35	24.1	85.5	189
26	9.37	18.0	0.11	1.18	1.60	2.21	0.43	7.14	4.36	17.3
27	10.9	22.9	0.17	0.91	1.93	30.4	0.76	10.3	23.1	90.1

28	9.56	17.4	0.10	0.71	2.02	2.57	0.67	9.56	2.98	30.7
29	8.52	18.9	0.11	0.86	1.46	2.77	0.62	10.5	4.37	38.1
30	21.6	45.9	0.24	0.74	15.0	42.0	1.06	18.2	40.8	148
31	33.1	60.6	0.36	1.40	12.9	31.0	3.23	25.6	47.3	172
32	41.1	68.0	0.45	1.01	33.4	60.6	2.75	23.7	70.3	172
33	49.8	88.8	0.56	1.26	14.4	30.0	2.43	20.6	118	224
34	20.0	30.3	0.34	0.89	14.9	33.5	1.38	19.6	38.5	116
35	10.7	13.8	0.39	0.79	4.09	10.7	1.65	13.5	34.2	72.8
36	27.4	46.9	0.47	1.18	11.6	22.7	2.33	16.4	63.8	130
37	12.3	19.2	0.21	0.32	6.02	6.22	1.09	11.4	18.1	35.6
38	9.44	15.3	0.11	1.08	3.90	4.22	1.16	8.66	9.5	23.1
39	9.27	15.1	0.28	1.01	3.10	5.55	1.75	12.4	10.6	56.8
40	52.3	61.7	0.68	1.35	11.6	23.2	3.94	19.3	90.5	144
41	25.4	44.8	0.42	0.87	18.4	36.7	1.61	14.4	58.3	124
42	13.6	19.4	0.19	0.34	6.01	14.9	2.87	16.0	18.6	54.9
43	16.5	18.6	0.32	1.42	5.72	6.90	2.37	7.95	32.1	42.2
44	20.4	247	1.32	2.80	12.6	39.6	7.63	39.9	183	505
45	4.05	11.6	0.08	1.28	0.77	2.45	1.20	11.1	6.23	15.9
46	12.2	19.8	0.25	0.87	4.13	7.35	4.39	16.8	14.8	63.7
47	14.6	21.8	0.25	1.63	7.65	12.1	2.75	13.2	23.6	55.5
48	12.8	23.0	0.14	1.25	3.95	6.17	2.42	11.9	7.24	32.6
49	34.4	43.6	1.01	2.07	16.3	22.3	6.09	20.5	105	115
50	6.76	20.3	0.08	0.71	0.97	1.77	0.73	8.09	3.07	20.8
51	9.15	18.6	0.15	0.69	2.54	5.99	1.42	10.7	9.97	40.3
52	162	244	1.29	1.55	41.8	58.8	6.52	22.5	204	230
53	20.0	24.0	0.43	0.96	6.13	11.8	3.55	16.2	203	284
54	10.4	24.8	0.25	1.95	2.54	5.30	1.39	13.5	13,9	56.0
55	10.4	20.9	0.30	0.50	4.53	11.6	2.96	19.6	12.0	51.2
56	11.7	31.4	0.22	2.69	5.21	12.7	2.00	13.8	17.3	77.4
57	9.54	29.5	0.18	1.17	3.19	9.59	1.25	13.6	14.2	41.1
58	19.9	90.2	0.46	0.56	7.27	16.8	2.40	20.2	30.7	95.1
59	18.3	29.2	0.49	1.37	9.83	25.3	5.92	33.5	49.3	106
60	15.7	19.8	0.32	1.27	11.9	23.7	2.69	10.0	33.7	44.5
61	22.6	44.7	0.29	0.97	6.00	17.2	2.15	16.3	66.0	156
62	14.0	44.1	0.16	1.65	3.65	19.2	1.21	22.9	11.4	38.5
63	14.7	29.3	0.20	0.94	5.18	11.1	1.34	18.2	13.8	80.3
64	18.5	28.3	0.13	1.11	3.68	6.34	0.74	10.4	3.36	40.2
65	12.4	24.7	0.13	1.06	2.15	7.70	0.64	12.0	5.10	35.5
66	16.8	22.8	0.28	1.11	6.31	8.29	1.44	11.2	21.2	49.4
67	15.9	55.8	0.23	1.06	3.97	17.7	0.90	24.3	20.3	85.8
68	22.7	29.1	0.28	0.78	7.62	12.3	0.73	11.9	20.9	72.5
69	8.23	26.1	0.15	1.52	3.25	22.3	1.21	25.4	13.5	95.4
70	10.0	20.0	0.14	0.28	3.24	8.27	0.53	10.9	17.8	47.2
71	13.3	22.3	0.14	0.93	7.31	11.1	0.59	11.0	12.7	63.3
72	23.6	39.0	0.57	0.94	6.92	14.0	3.16	21.0	208	343
73	23.6	26.9	0.39	0.77	13.5	18.5	2.42	16.1	87.4	99.0
74	32.4	47.0	0.51	1.18	13.2	25.0	3.00	17.3	119	280
75	38.7	56.4	0.48	1.47	14.1	26.8	4.13	24.4	60.9	134
76	18.4	27.1	0.32	0.86	9.96	11.3	3.08	13.6	28.4	53.8
77	32.0	40.1	0.13	1.15	2.22	15.1	0.43	19.8	9.00	75.0
78	20.4	24.8	0.33	1.15	5.60	7.39	1.52	11.1	91.6	139

Table S3. Contamination factors (CF) and pollution load index (PLI).

No	CF Pb	CF Cd	CF Cu	CF Ni	CF Zn	PLI	No	CF Pb	CF Cd	CF Cu	CF Ni	CF Zn	PLI
1	0.8	0.3	0.4	0.2	0.2	0.32	26	0.9	0.2	0.5	0.1	0.2	0.31
2	4.9	1.5	16.1	1.3	2.6	3.32	27	1.1	0.3	0.6	0.3	0.9	0.56
3	1.6	0.4	1.2	0.2	0.6	0.64	28	1.0	0.2	0.7	0.2	0.1	0.32
4	10.3	4.2	5.5	2.1	14.3	5.88	29	0.9	0.2	0.5	0.2	0.2	0.32
5	1.6	0.4	2.0	0.3	0.5	0.75	30	2.2	0.5	5.0	0.4	1.6	1.24
6	3.3	1.2	5.8	1.0	2.7	2.29	31	3.3	0.7	4.3	1.1	1.9	1.84
7	1.0	0.3	0.8	1.2	0.2	0.60	32	4.1	0.9	11.1	0.9	2.8	2.54
8	1.4	0.4	0.8	0.7	0.2	0.56	33	5.0	1.1	4.8	0.8	4.7	2.53
9	1.6	0.4	1.0	0.3	0.2	0.56	34	2.0	0.7	5.0	0.5	1.5	1.37
10	1.4	0.4	1.0	0.3	0.6	0.65	35	1.1	0.8	1.4	0.6	1.4	0.97
11	2.5	0.5	2.7	0.6	0.5	0.98	36	2.7	0.9	3.9	0.8	2.6	1.82
12	1.3	0.5	1.8	0.4	0.5	0.72	37	1.2	0.4	2.0	0.4	0.7	0.77
13	1.3	0.4	1.1	0.3	0.5	0.61	38	0.9	0.2	1.3	0.4	0.4	0.52
14	1.1	0.3	1.1	0.4	0.2	0.52	39	0.9	0.6	1.0	0.6	0.4	0.67
15	1.4	0.5	1.4	0.5	0.4	0.70	40	5.2	1.4	3.9	1.3	3.6	2.65
16	0.9	0.2	0.9	0.3	0.4	0.45	41	2.5	0.8	6.1	0.5	2.3	1.75
17	0.9	0.2	0.5	0.2	0.2	0.32	42	1.4	0.4	2.0	1.0	0.7	0.94
18	1.7	0.3	1.2	0.4	0.6	0.69	43	1.7	0.6	1.9	0.8	1.3	1.15
19	0.9	0.3	1.1	0.6	0.5	0.60	44	2.0	2.6	4.2	2.5	7.3	3.35
20	3.6	1.1	7.6	1.6	3.3	2.78	45	0.4	0.2	0.3	0.4	0.2	0.28
21	0.7	0.3	0.4	0.2	0.2	0.29	46	1.2	0.5	1.4	1.5	0.6	0.94
22	1.2	0.4	1.4	0.3	0.6	0.66	47	1.5	0.5	2.6	0.9	0.9	1.10
23	1.0	0.1	0.8	0.1	0.1	0.24	48	1.3	0.3	1.3	0.8	0.3	0.64
24	4.5	1.5	8.2	1.4	2.7	2.90	49	3.4	2.0	5.4	2.0	4.2	3.17
25	4.1	1.3	10.8	1.1	3.4	2.93	50	0.7	0.2	0.3	0.2	0.1	0.25
51	0.9	0.3	0.8	0.5	0.4	0.54	65	1.2	0.3	0.7	0.2	0.2	0.40
52	16.3	2.6	13.9	2.2	8.2	6.36	66	1.7	0.6	2.1	0.5	0.8	0.96
53	2.0	0.9	2.0	1.2	8.1	2.02	67	1.6	0.5	1.3	0.3	0.8	0.75
54	1.0	0.5	0.8	0.5	0.6	0.65	68	2.3	0.6	2.5	0.2	0.8	0.92
55	1.0	0.6	1.5	1.0	0.5	0.85	69	0.8	0.3	1.1	0.4	0.5	0.57
56	1.2	0.4	1.7	0.7	0.7	0.84	70	1.0	0.3	1.1	0.2	0.7	0.52
57	1.0	0.4	1.1	0.4	0.6	0.61	71	1.3	0.3	2.4	0.2	0.5	0.62
58	2.0	0.9	2.4	0.8	1.2	1.34	72	2.4	1.1	2.3	1.1	8.3	2.22
59	1.8	1.0	3.3	2.0	2.0	1.87	73	2.4	0.8	4.5	0.8	3.5	1.88
60	1.6	0.6	4.0	0.9	1.3	1.37	74	3.2	1.0	4.4	1.0	4.8	2.33
61	2.3	0.6	2.0	0.7	2.6	1.38	75	3.9	1.0	4.7	1.4	2.4	2.26
62	1.4	0.3	1.2	0.4	0.5	0.63	76	1.8	0.6	3.3	1.0	1.1	1.35
63	1.5	0.4	1.7	0.4	0.6	0.76	77	3.2	0.3	0.7	0.1	0.4	0.50
64	1.8	0.3	1.2	0.2	0.1	0.46	78	2.0	0.7	1.9	0.5	3.7	1.36

where: $CF = CF_{heavy\ metal}/CF_{background}$

CF_b – background levels for anthropogenic fraction of metals are:
0.5 mg/kg (Cd); 10 mg/kg (Pb); 3 mg/kg (Ni, Cu); 25 mg/kg (Zn) [56]

CF [59]:

1 < CF	low contamination;
1 ≤ CF < 3	moderate contamination;
3 ≤ CF < 6	considerable contamination;
CF ≥ 6	very high contamination

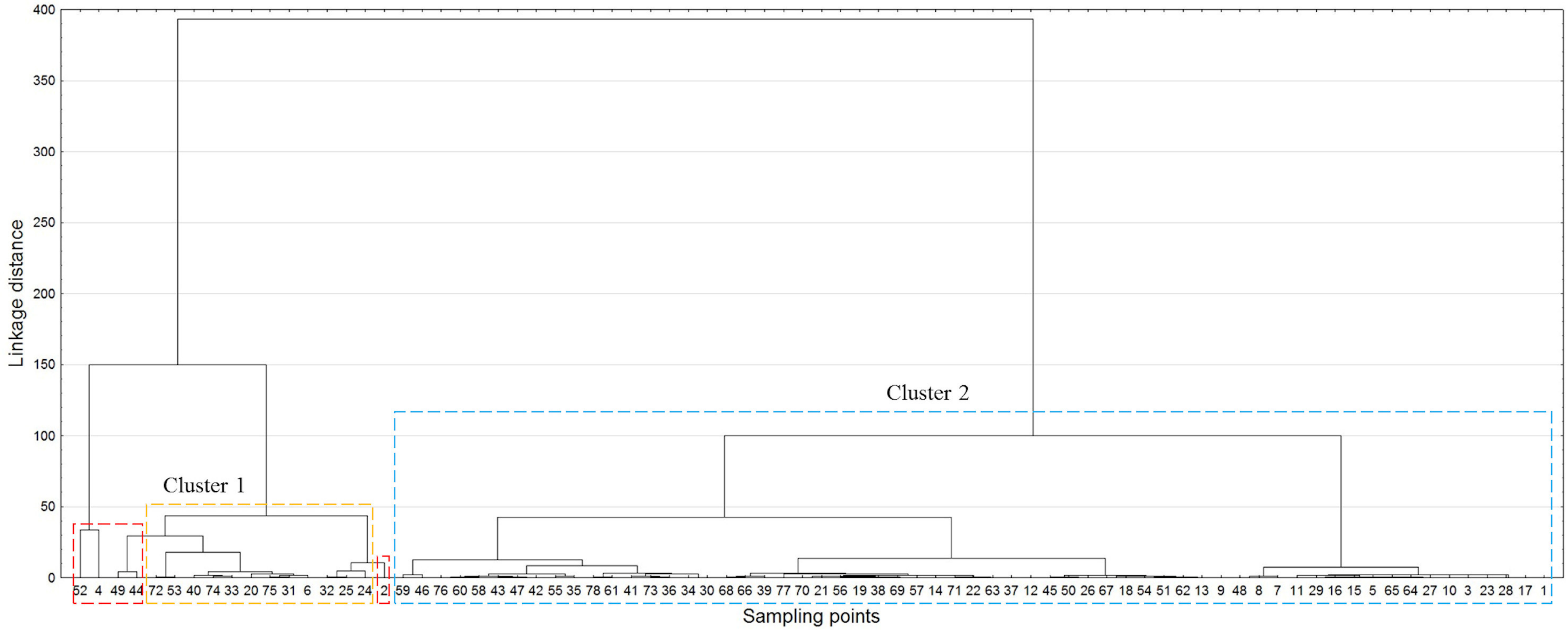

Figure S2. Dendrogram for sampling sites.

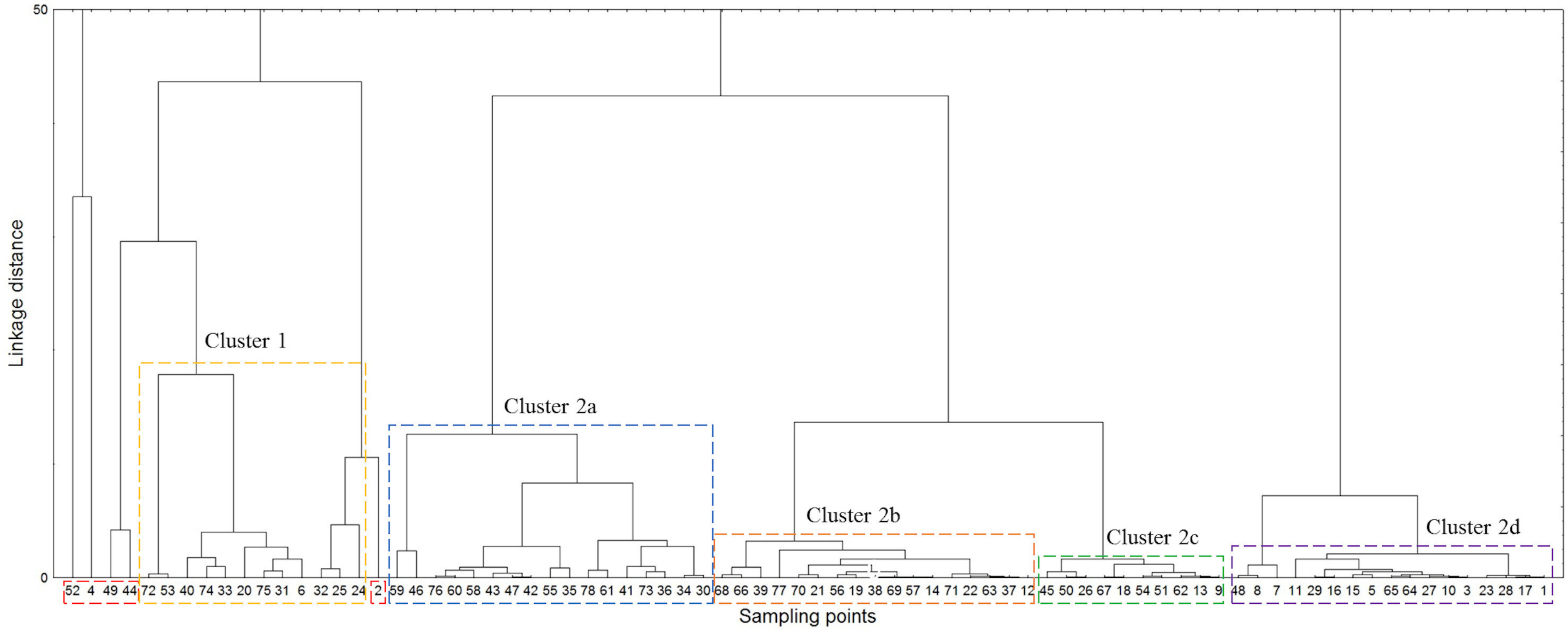

Figure S3. Dendrogram for sampling sites with scale of the linkage distance reducing to 50.

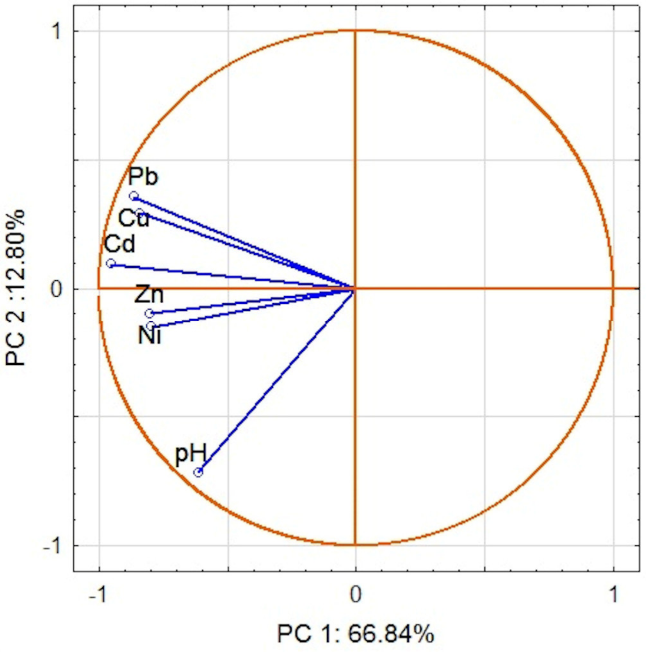

Figure S4. Loading plot of the two main components PC1 and PC2 for PCA without outliers.

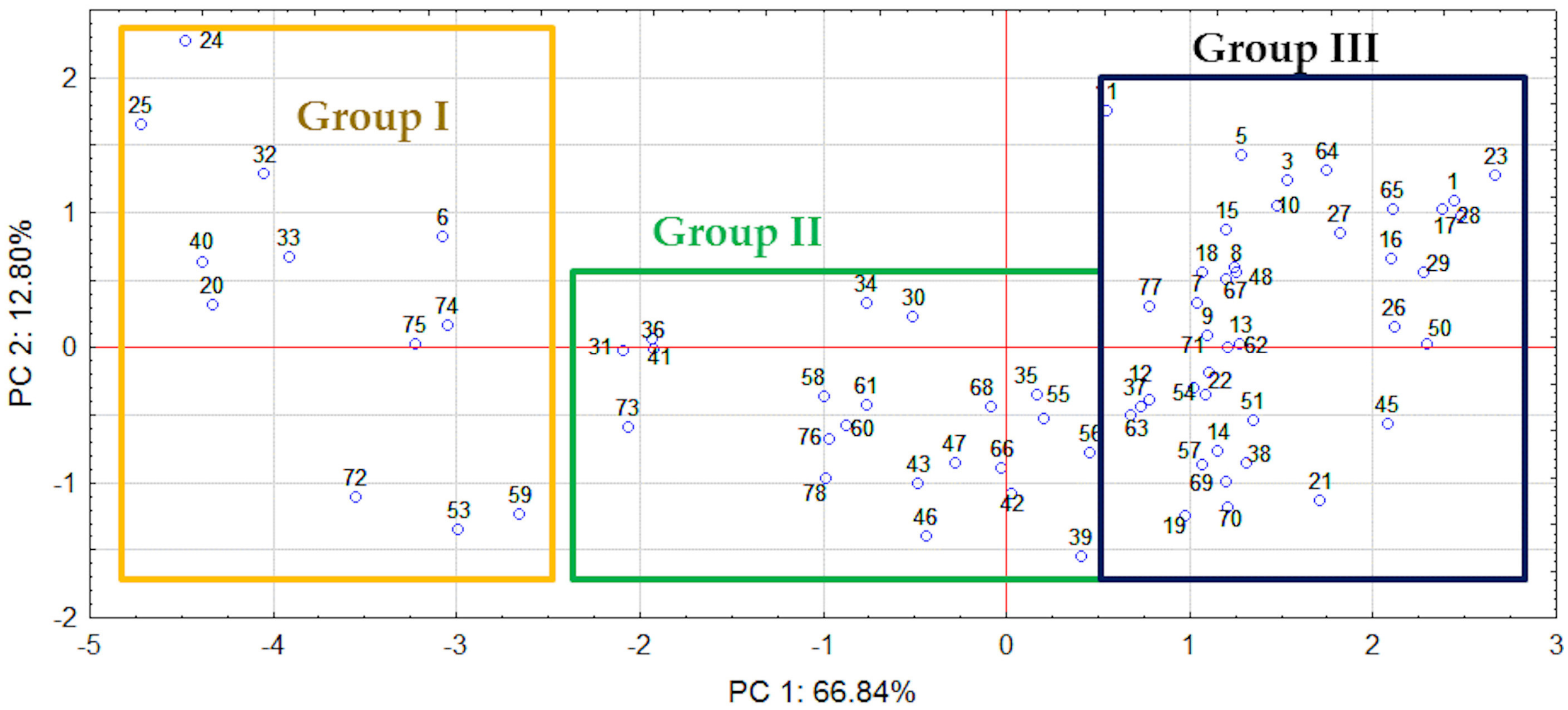

Figure S5. Score plot of PC1 versus PC2 for PCA without outliers.
